# Classification of cystoid macular edema status from retinal vasculitis fluorescein angiography images using a convolutional neural network

**DOI:** 10.3389/fopht.2026.1901237

**Published:** 2026-10-07

**Authors:** Celina Wang, Koushani Chakrabarty, Stephen D. Anesi, Peter Y. Chang

**Affiliations:** 1 Milton Academy, Milton, MA, United States; 2 Massachusetts Eye Research and Surgery Institution, Waltham, MA, United States; 3 Department of Biomedical Engineering, University at Buffalo, Buffalo, NY, United States

**Keywords:** convolutional neural network (CNN), cystoid macular edema (CME), fluorescein angiography, grad-cam, machine learning, patient-level cross-validation, retinal vasculitis, transfer learning

## Abstract

**Introduction:**

Cystoid macular edema (CME) is a vision-threatening complication of retinal vasculitis, a rare inflammatory disease for which large imaging datasets are difficult to obtain. Detection of CME on fluorescein angiography (FA) requires expert interpretation, motivating automated image-based approaches to support clinical decision-making. Deep learning methods, particularly convolutional neural networks (CNNs), have shown strong performance in medical image analysis but are constrained by limited labeled data in rare diseases. Transfer learning may mitigate this limitation by adapting pretrained models to small, task-specific datasets.

**Methods:**

We analyzed 207 de-identified late-phase 55° FA images from 117 patients with retinal vasculitis. The primary target was concurrent patient-level CME status, established through specialist review of clinical records and optical coherence tomography obtained at the same clinical visit as FA. A patient was classified as CME-positive when CME was present in at least one eye. An ImageNet-pretrained ResNet-18 was evaluated using five-fold nested, patient-level cross-validation. Within each outer-development cohort, 144 candidate configurations were compared by four-fold inner cross-validation and selected according to mean inner-validation AUROC. Each selected configuration was evaluated only on its corresponding untouched outer-test fold.

**Results:**

Across 117 pooled patient-level outer-test predictions, the model-selection pipeline achieved an AUROC of 0.708 (95% CI, 0.610–0.802), an AUPRC of 0.626 (95% CI, 0.493–0.755), and an accuracy of 0.726 (95% CI, 0.641–0.803). Sensitivity was 0.694 (95% CI, 0.558–0.822), specificity was 0.750 (95% CI, 0.643–0.853), and the F1 score was 0.680 (95% CI, 0.563–0.780). Exploratory Grad-CAM analysis visualized regions associated with model predictions.

**Discussion:**

The nested model-selection pipeline showed moderate discrimination with a relatively balanced operating profile and slightly higher specificity than sensitivity. Clinically relevant false-positive and false-negative errors, together with the absence of external validation, preclude clinical deployment.

## 1 Introduction

Cystoid macular edema (CME) is a leading cause of visual loss in patients with retinal vasculitis. In retinal vasculitis, inflammation-induced breakdown of the blood–retina barrier leads to vascular leakage and accumulation of intraretinal fluid, often manifesting as CME (1). If left untreated, chronic CME can result in irreversible retinal damage and permanent vision loss. Because angiographic manifestations may be subtle and require careful specialist interpretation, timely recognition of CME-associated findings can be challenging. Accurate identification is clinically important because the presence of CME may influence treatment and monitoring decisions.

Diagnosis of CME in retinal vasculitis relies on clinical examination and retinal imaging modalities such as optical coherence tomography (OCT) and fluorescein angiography (FA). Not all patients with retinal vasculitis develop CME, and its presence may influence decisions regarding treatment initiation or escalation. Identifying angiographic features associated with CME could therefore support more consistent image interpretation and help prioritize cases requiring further clinical assessment. OCT provides direct structural evidence of intraretinal fluid and serves as an important component of the clinical reference standard, whereas FA depicts vascular leakage and inflammatory activity that may be associated with CME. FA is widely used to visualize retinal vascular leakage and macular involvement, particularly in inflammatory retinal disease (2). Based on the clinical experience of the authors specializing in uveitis and ocular inflammatory disease (SDA and PYC), FA is an important component of retinal vasculitis assessment and may inform decisions regarding initiation or escalation of local and systemic therapy. Three angiographic findings were considered potentially associated with CME: para- and perifoveal hyperfluorescence within the temporal vascular arcades, diffuse punctate hyperfluorescence outside the macular region, and late-phase optic disc hyperfluorescence. However, interpretation of FA images requires specialized expertise and is time-consuming, as clinicians must manually assess late-phase leakage patterns suggestive of edema. This process is inherently subjective and may be inconsistent across observers or clinical settings, especially in resource-limited environments where access to experienced retinal specialists is restricted. An automated approach for analyzing FA images and identifying angiographic patterns associated with CME could therefore support more consistent FA interpretation and clinical review.

Recent advances in artificial intelligence (AI), particularly in computer vision, have demonstrated that deep learning models can identify subtle visual patterns in medical images with high accuracy (3). Convolutional neural networks (CNNs) have demonstrated strong performance in several ophthalmic applications, including diabetic retinopathy screening from fundus photographs (4) and disease classification from OCT images (5). These successes highlight the ability of CNNs to learn clinically meaningful features directly from raw image data, without reliance on handcrafted rules. Nonetheless, most prior work has focused on common retinal diseases with access to large annotated datasets, while applications to rare inflammatory conditions such as retinal vasculitis remain limited. Although recent studies have explored deep learning-based analysis of FA images for tasks such as vascular leakage and occlusion segmentation, automated identification of CME from FA images in retinal vasculitis has received limited investigation (6).

A major challenge in applying deep learning to rare diseases is data scarcity. Training CNNs from scratch typically requires large, diverse datasets to ensure robust generalization, which is often infeasible for uncommon conditions. Transfer learning has emerged as an effective strategy to address this limitation by reusing feature representations learned from large-scale datasets and adapting them to smaller, taskspecific medical imaging problems through fine-tuning (7). This approach has been widely adopted in medical image analysis and has shown improved performance and reduced overfitting in data-limited settings.

In this study, we developed and evaluated a transfer learning-based framework for identifying angiographic patterns associated with concurrent CME from late-phase FA images. We hypothesized that a convolutional neural network could learn angiographic patterns associated with CME, including macular and extramacular hyperfluorescence and optic disc leakage, and distinguish CME-positive from CME-negative patients. Given the limited rare-disease cohort, performance was evaluated using nested patient-level cross-validation. Configuration selection was conducted exclusively within each outerdevelopment cohort, and the corresponding outer-test fold remained untouched until final evaluation. The primary contribution of this proof-of-concept study is the leakage-controlled evaluation of a complete model-selection pipeline for FA-based CME classification, rather than the development of a new network architecture or a single finalized clinical model.

## 2 Materials and methods

This study received approval from the New England Institutional Review Board (IRB) before the data collection process began (WCG IRB tracking ID: 120160012). All tenets of the Declaration of Helsinki were followed, and all compliance rules of the US Health Insurance Portability and Accountability Act (HIPAA) of 1996 were respected. The requirement for informed consent was waived by the IRB; parental permission and assent were also waived for minors. Separate consent was obtained from patients whose representative clinical images are featured in the manuscript.

### 2.1 Model architecture and transfer learning

Deep learning has become a dominant approach for image-based classification tasks due to its ability to learn hierarchical feature representations directly from raw pixel data. In particular, convolutional neural networks (CNNs) have demonstrated strong performance across a wide range of computer vision problems, including object recognition, medical image classification, and disease detection. CNNs are especially well suited for medical imaging applications because they can capture both low-level features, such as edges and intensity gradients, and higher-level spatial patterns relevant to pathology, without requiring handcrafted feature engineering.

Among CNN architectures, residual networks (ResNets) have been widely adopted due to their ability to train deeper models efficiently through the use of residual (skip) connections (8). These connections mitigate vanishing gradient issues and enable effective feature reuse across layers, leading to improved convergence and generalization. ResNet architectures have been successfully applied in ophthalmology for tasks such as diabetic retinopathy grading (9), age-related macular degeneration detection (10), and retinal image analysis (11), demonstrating robustness across diverse imaging modalities.

A major limitation of deep learning in medical imaging is the requirement for large labeled datasets, which are often unavailable for rare diseases such as retinal vasculitis. Training deep CNNs from scratch on small datasets can lead to overfitting and poor generalization. Transfer learning addresses this challenge by initializing models with weights pretrained on large-scale datasets, such as ImageNet, which contain millions of natural images spanning diverse visual categories. Although natural images differ from medical images, pretrained networks learn general-purpose visual features—such as edges, textures, and spatial compositions—that are transferable to medical imaging tasks. Fine-tuning these pretrained models on domain-specific datasets has been shown to significantly improve performance and stability in data-limited settings (12).

In this study, we employed a ResNet-based CNN initialized with ImageNet-pretrained weights and adapted it for binary classification of concurrent cystoid macular edema (CME) from fluorescein angiography images. A ResNet-18 architecture was selected to balance representational capacity and computational efficiency, which was particularly important given the modest dataset size. The original classification head was replaced with a dropout layer (*p* = 0.5) followed by a fully connected linear layer producing two logits representing CME-negative and CME-positive evidence for each image. FA images were converted to single-channel grayscale, and the first convolutional layer of ResNet-18 was modified accordingly to accept a single-channel input. The pretrained ImageNet weights for this layer were averaged across the three RGB input channels to initialize the corresponding grayscale filters. All remaining pretrained backbone parameters were retained, whereas the task-specific classification head was initialized *de novo*.

For each patient, all available eye images were treated as a variable-size multiple-instance learning bag and processed by the same shared ResNet-18 encoder. Image-level CME log-odds were calculated as the difference between the CME-positive and CME-negative logits. The maximum image-level CME log-odds across all available images was then used as the patient-level score, consistent with the prespecified clinical rule that a patient was classified as CME-positive when CME was present in at least one eye. The patient-level log-odds were converted into CME and non-CME probabilities using a two-class softmax representation. Training and evaluation therefore produced one label, one contribution to the training loss, one CME probability, and one final prediction per patient, rather than independent final predictions for individual eye images.

The complete experimental workflow, including dataset preparation, preprocessing, nested patient-level cross-validation, transfer learning, threshold selection, performance evaluation, explainability analysis, and nested hyperparameter analysis, is summarized in **Figure 1**.

**Figure 1:**
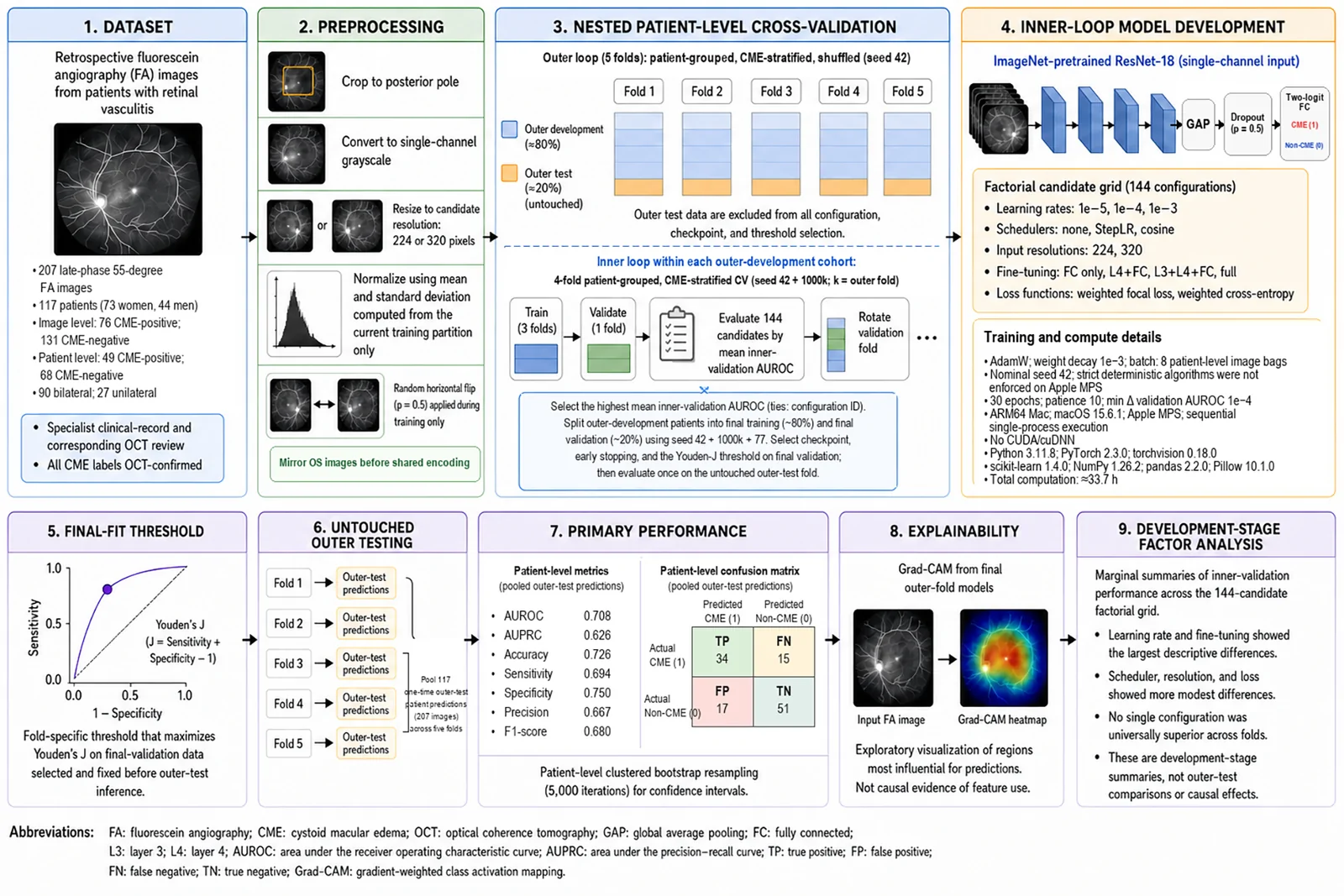
Overview of the nested deep learning framework for CME classification from fluorescein angiography.

The transfer learning–based approach enables effective utilization of limited clinical data while retaining the expressive power of deep neural architectures, making it well suited for rare-disease imaging applications such as retinal vasculitis.

### 2.2 Dataset and data preprocessing

This study utilized a retrospective dataset of fluorescein angiography (FA) images from patients diagnosed with idiopathic retinal vasculitis. Images consisted of fluorescein angiography obtained approximately 3 minutes after fluorescein injection using the Heidelberg Spectralis imaging system. The finalized analytic dataset included 207 de-identified late-phase FA images with a 55°field of view from 117 unique patients, including 73 women and 44 men. 90 patients contributed images from both eyes, while 27 contributed an image from only one eye; among the latter, 13 contributed right-eye images and 14 contributed left-eye images. At the image level, the dataset contained 76 CME-positive and 131 non-CME images. At the primary patient level, patients were classified as CME-positive when CME was present in at least one eye, resulting in 49 CME-positive and 68 CME-negative patients. Five CME-positive patients had one CME-positive eye and one non-CME eye. Patients were excluded if CME onset preceded the diagnosis of retinal vasculitis, because the CME could not be attributed to the clinical course of retinal vasculitis. Cohort selection and exclusions are summarized in **Figure 2**, and the cohort age distribution is reported in **Table 1**.

**Figure 2:**
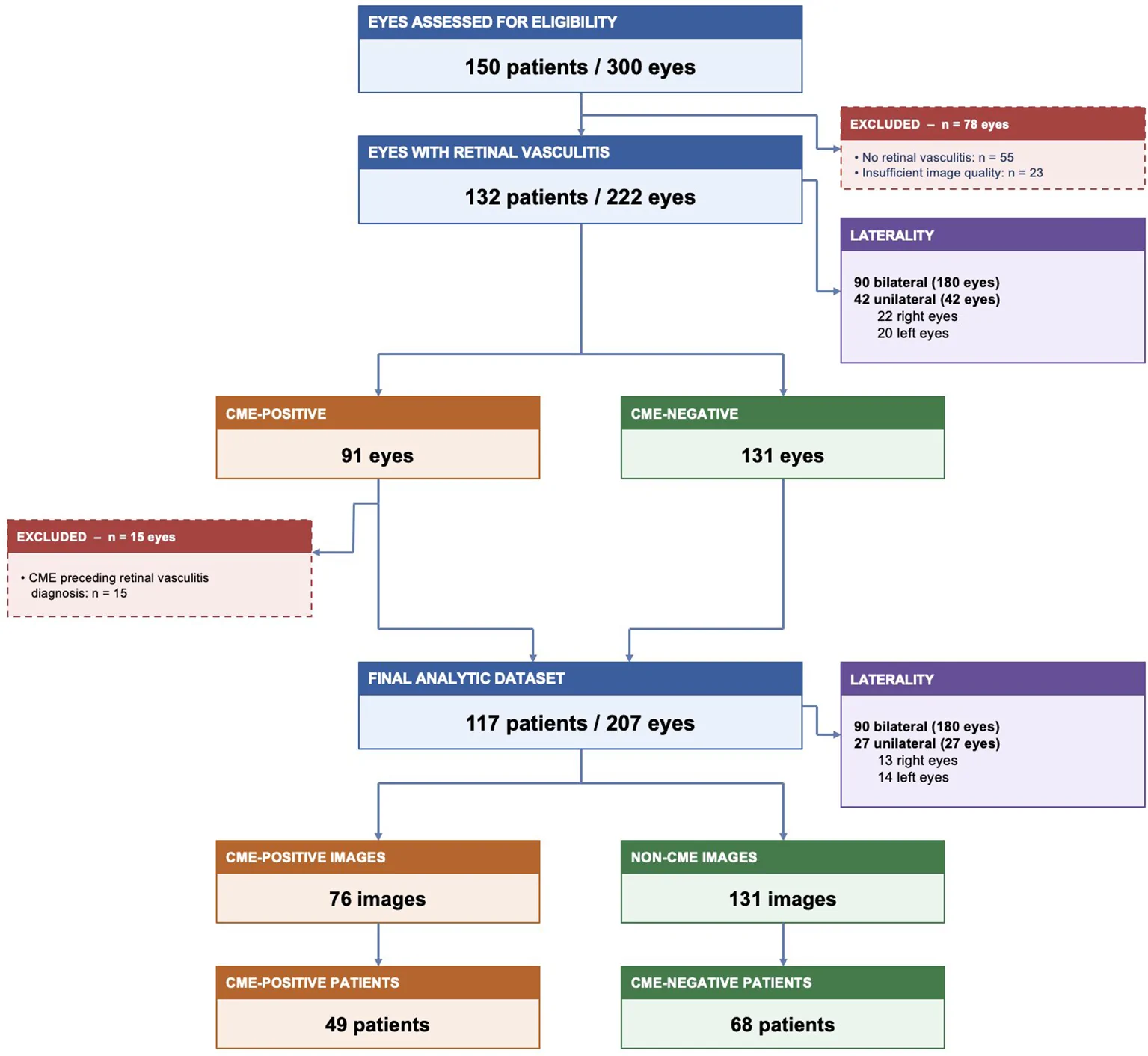
Study cohort selection and final analytic dataset. Of 300 eyes assessed, 207 images from 117 patients were included, comprising 76 CME-positive and 131 non-CME images.

**Table 1:** Age distribution of the study cohort.

Age group (years)	n	Percentage
*<*18	3	2.6%
18–29	16	13.7%
30–39	23	19.7%
40–49	12	10.3%
50–59	26	22.2%
60–69	20	17.1%
70–79	8	6.8%
≥80	9	7.7%
Total	117	100%

Ground-truth CME status was established through retrospective expert review of clinical records and optical coherence tomography (OCT) obtained at the same clinical visit as the fluorescein angiography examination. The primary outcome was concurrent patient-level CME status. A patient was classified as CME-positive when CME was present in at least one eye. Accordingly, patients with unilateral CME were classified as CME-positive, and all available FA images from each patient were modeled together as a single multiple-instance learning bag. Patients in the non-CME group had no CME in either eye at the index FA/OCT visit. Apart from the separately described exclusion of 15 patients whose CME onset preceded the diagnosis of retinal vasculitis, absence of CME at other time points was not an eligibility requirement for the non-CME group. Review of the available clinical records nevertheless showed that none of the patients ultimately classified as CME-negative had documented CME at another time. This study did not evaluate incident or future CME development.

Images with JPEG, JPG, or PNG extensions were loaded using Pillow and converted to single-channel grayscale with Image.convert(“L”). For each model fit, images were resized to the candidate resolution of 224 or 320 pixels, converted to tensors, and normalized using statistics calculated exclusively from the corresponding training partition. Random horizontal flipping with probability 0.5 was applied only to training images; validation and test transformations contained no stochastic augmentation. Left-eye (OS) images were mirrored before shared encoding to standardize laterality. Normalization statistics were calculated after resizing and scaling pixel intensities by 1*/*255. The mean was calculated across all pixels from all images in the current training partition, and the population standard deviation was calculated as $\sqrt{E\left[x^{2}\right]-E{\left[x\right]}^{2}}$, with variance bounded below by 10^−12^. These training-derived values were applied unchanged to the corresponding validation partition and, for final outer-fold fits, to the outer-test partition. Statistics were recomputed for every distinct training partition and input resolution. Representative examples of late-phase fluorescein angiography images of retinal vasculitis patients, including CME-positive and CME-negative cases, are presented in **Figure 3** to illustrate the angiographic features used for model training. **Figure 4** provides an anatomical reference of a normal retina highlighting key structures. Posteriorpole cropping was performed during dataset preparation before images entered the released analytic pipeline. The cropped region encompassed the macula, optic disc, and surrounding retinal vasculature, as illustrated by the orange box in **Figure 4**. The released modeling code therefore begins from the previously cropped images and performs grayscale conversion, laterality alignment, resizing, augmentation, and training-partition normalization.

**Figure 3:**
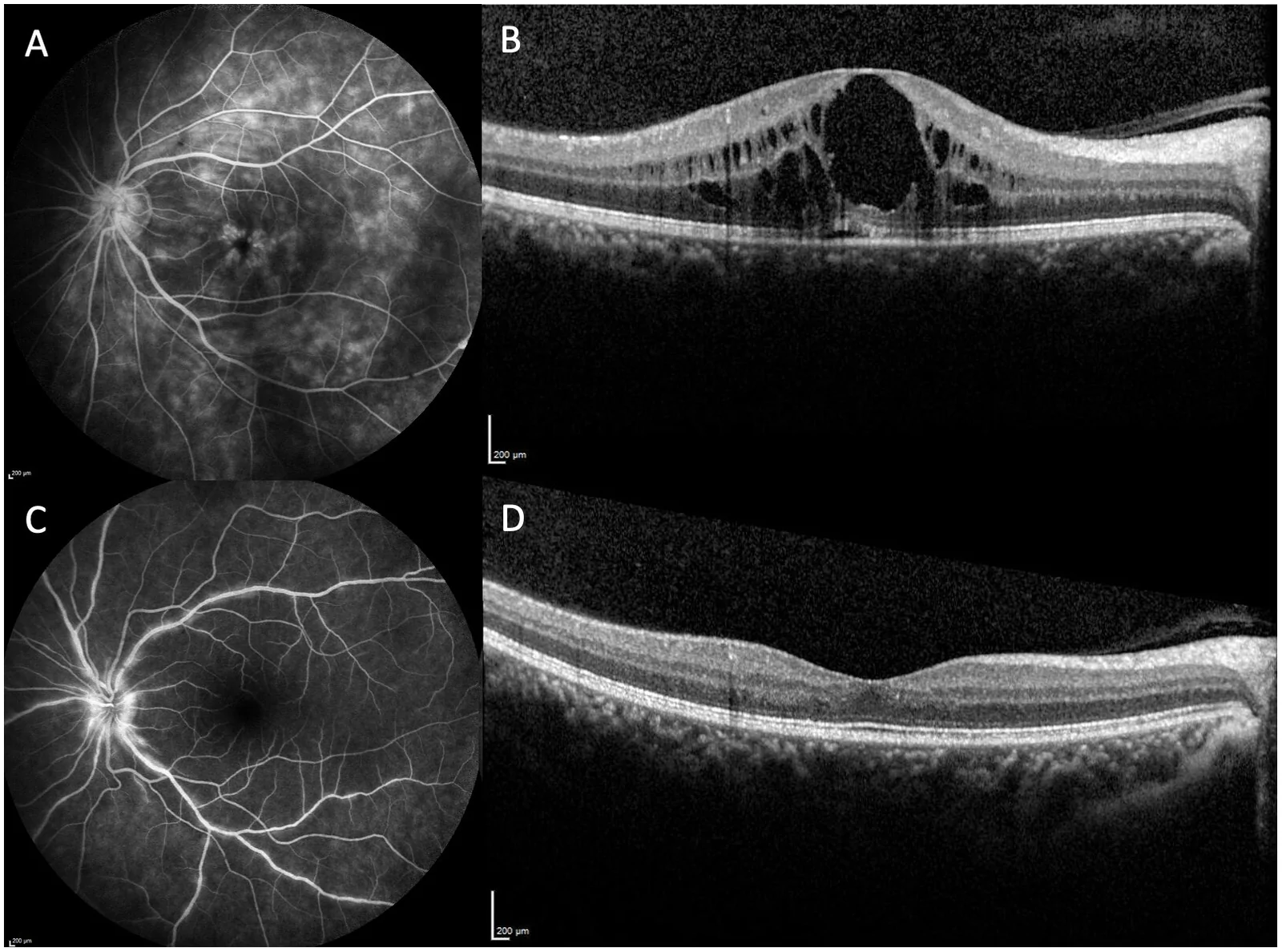
Representative same-visit late-phase fluorescein angiography (FA) and optical coherence tomography (OCT) B-scan pairs from eyes with retinal vasculitis with and without cystoid macular edema (CME). Panels **(A, B)** depict an eye with CME, demonstrating petalloid macular leakage and increased optic disc leakage on FA with corresponding intraretinal cystic spaces on the OCT B-scan. Panels **(C, D)** depict an eye without CME, with preserved foveal contour and no intraretinal fluid on the OCT B-scan.

**Figure 4:**
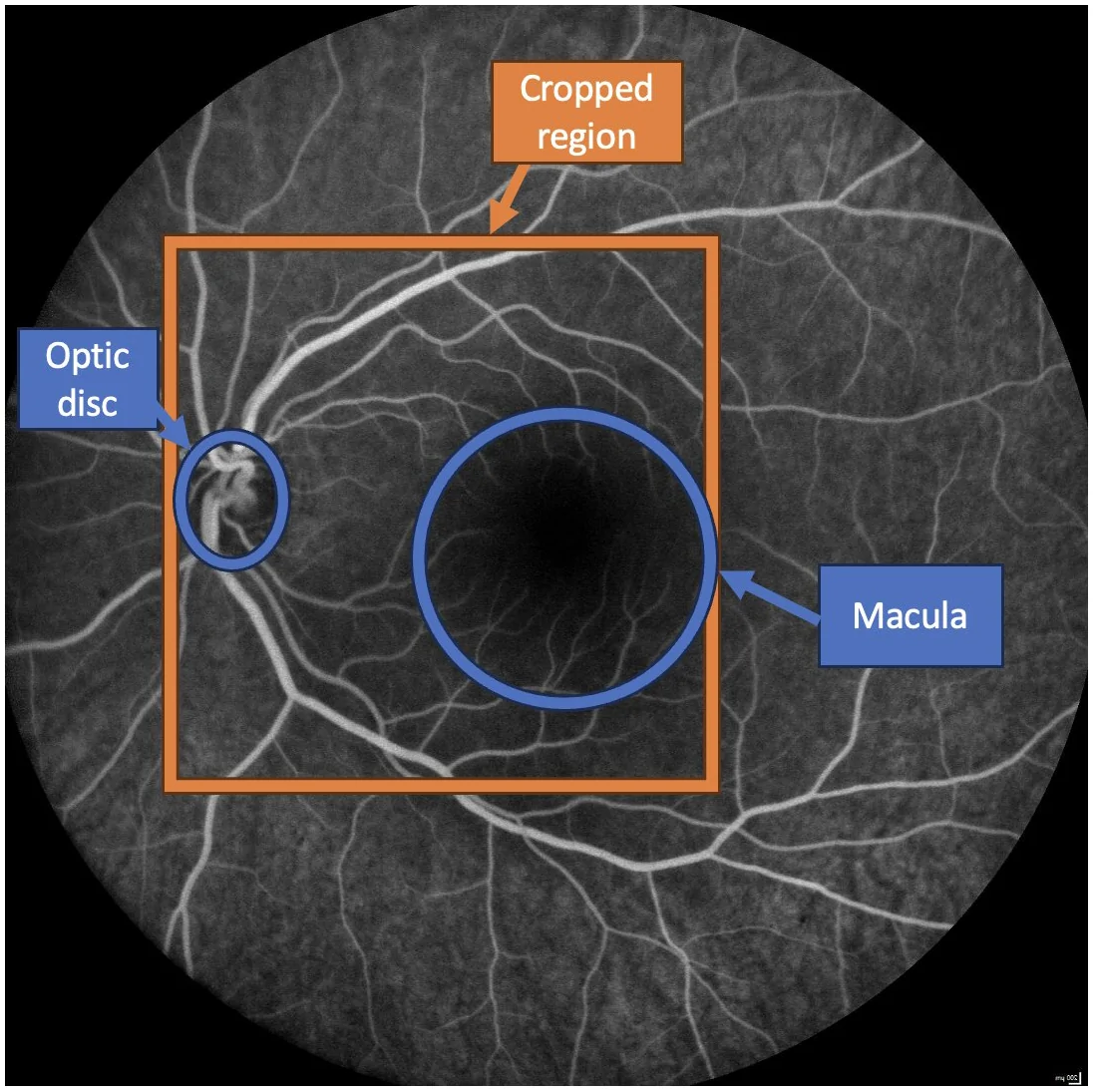
Labeled fluorescein angiography of a normal retina (OS) without evidence of retinal vascular leakage. This representative image was obtained from the Massachusetts Eye Research and Surgery Institution outside the study cohort and is shown for anatomical reference only.

The exact implementation used for the final patient-level analysis, including data preprocessing, multipleinstance learning, training-partition normalization, nested cross-validation, validation-derived threshold selection, Grad-CAM, and hyperparameter analysis, is available at the fixed repository commit cited below 1.

### 2.3 Training

Model development and evaluation used five-fold nested cross-validation with patient-level grouping. The outer loop used StratifiedGroupKFold implemented in scikit-learn version 1.4.0, with grouping by patient identifier, stratification by the derived patient-level CME label, shuffling, and a random seed of 42. In each outer iteration, approximately one fifth of patients formed an untouched outer-test set and the remaining patients formed the outer-development set. Configuration selection was performed only within the outer-development set using four-fold StratifiedGroupKFold. Source eye-level labels^1^ were retained for cohort description and aggregated according to the prespecified patient-level rule: a patient was classified as CME-positive when at least one available eye was CME-positive. Five patients had discordant eye-level labels. After this aggregation, every image within a patient bag was assigned the same derived patient label, which was used for stratification, training, and evaluation.

The candidate set comprised 144 prespecified combinations of learning rate (1 × 10^−5^, 1 × 10^−4^, or 1 × 10^−3^), scheduler (none, step decay, or cosine annealing), input resolution (224 or 320 pixels), finetuning depth (classifier only, layer4+classifier, layer3+layer4+classifier, or full network), and loss (weighted cross-entropy or weighted focal loss). Candidates were ranked by mean four-fold inner-validation AUROC. Inner split seeds were 42 + 1000*k*, where *k* denotes the outer-fold number. Outer-test AUROC was not used to rank candidates or resolve ties; exact ties were resolved by lexicographically ascending configuration identifier. Different configurations could therefore be selected in different outer folds.

After configuration selection, the outer-development cohort was split using the first partition from a shuffled, five-fold patient-level StratifiedGroupKFold. This produced a final-training subset of approximately 80% and a final-validation subset of approximately 20% of the outer-development patients. The final-validation seeds were 42 + 1000*k* + 77, where *k* denotes the outer-fold number. Checkpoint selection and early stopping used validation AUROC, with patience 10 and minimum improvement 1×10^−4^. The fold-specific operating threshold was selected on this same final-validation subset by maximizing Youden’s J statistic. The selected checkpoint and threshold were then evaluated once on the untouched outertest set. No outer-test patient, image, label, prediction, or performance metric was used for configuration, checkpoint, or threshold selection.

Images were converted to single-channel grayscale tensors and resized according to the candidate configuration. Left-eye (OS) images were mirrored before shared encoding to standardize laterality. Random horizontal flipping with probability 0.5 was applied only during training. Validation and test transformations contained no stochastic augmentation. The normalization mean and population standard deviation were calculated exclusively from the applicable training partition and recomputed for every distinct training partition and input resolution.

Class weights were calculated from the patient-level class distribution in each training partition. For class *c*, the weight was defined as


$$w_{c}=\frac{N}{2n_{c}},$$

where *N* is the total number of patients in the applicable training partition and *n_c_* is the number of patients belonging to class *c*. These training-derived weights were used for both weighted cross-entropy and class-weighted focal loss. The focal-loss focusing parameter was *γ* = 2.0.

Optimization used AdamW with a weight decay of 1 × 10^−3^, a batch size of eight patient bags, and a maximum of 30 epochs. Step decay, when selected, used a step size of 10 epochs and a multiplicative factor of 0.5. Cosine annealing used *T*_max_ = 30 and a minimum learning rate of 10^−6^; the third scheduler condition applied no learning-rate scheduler. Checkpoint selection monitored validation AUROC. A checkpoint was saved when validation AUROC improved by more than 1×10^−4^, and training stopped after 10 consecutive epochs without such an improvement. Training batches were shuffled, whereas validation and test batches were not.

The finalized analysis was executed sequentially in a single process using Apple Metal Performance Shaders (MPS) on an ARM64 Mac running macOS 15.6.1. The software environment comprised Python 3.11.8, PyTorch 2.3.0, torchvision 0.18.0, scikit-learn 1.4.0, NumPy 1.26.2, pandas 2.2.0 and Pillow 10.1.0. CUDA and cuDNN were not used.

Patient and image counts for the final-training, final-validation, and outer-test partitions are reported in **Table 2**.

**Table 2:** Patient and image counts for the final training, final validation, and untouched outer-test subsets in each nested cross-validation fold.

Fold	Final training		Final validation		Outer test	
	Patients	Images	Patients	Images	Patients	Images
1	74 (29/45)	133 (50/83)	19 (5/14)	34 (8/26)	24 (15/9)	40 (23/17)
2	74 (31/43)	131 (50/81)	19 (9/10)	33 (16/17)	24 (9/15)	43 (15/28)
3	75 (34/41)	132 (54/78)	19 (6/13)	34 (11/23)	23 (9/14)	41 (16/25)
4	75 (32/43)	135 (54/81)	19 (8/11)	31 (11/20)	23 (9/14)	41 (16/25)
5	75 (31/44)	133 (53/80)	19 (11/8)	32 (17/15)	23 (7/16)	42 (11/31)

Parentheses indicate CME-positive/CME-negative counts, respectively. Patient counts use the primary patient-level endpoint. Image counts reflect the patient-level label assigned to each image after within-patient aggregation and therefore differ from the source eye-level counts.

### 2.4 Evaluation and metrics

Primary performance was evaluated using the five untouched outer-test folds of the nested patient-level cross-validation framework. In each outer iteration, configuration selection was completed using only fourfold inner cross-validation within the outer-development cohort. After configuration selection, checkpoint selection, early stopping, and operating-threshold selection were performed using the corresponding finalvalidation subset. The fold-specific operating threshold was selected by maximizing Youden’s J statistic and fixed before evaluation on the corresponding outer-test fold. Each patient received exactly one prediction from the fold-specific model, and each patient appeared in an outer-test fold exactly once. Predictions from the five outer-test folds were pooled to evaluate the complete model-selection pipeline rather than a single fixed model configuration.

To comprehensively assess model performance, both threshold-independent and threshold-dependent metrics were reported. Threshold-independent metrics included the area under the receiver operating characteristic curve (AUROC) and the area under the precision–recall curve (AUPRC). AUROC quantifies the model’s ability to distinguish CME-positive from CME-negative patients independently of any specific classification threshold, whereas AUPRC summarizes the precision–recall trade-off and is particularly informative for imbalanced medical datasets because it emphasizes performance on the positive class.

Threshold-dependent performance was evaluated using accuracy, sensitivity, specificity, precision, and F1score. Accuracy summarizes overall classification performance, while sensitivity and specificity quantify the proportions of CME-positive and CME-negative patients correctly classified, respectively. Sensitivity is particularly important because missed CME cases may delay diagnosis and treatment, whereas specificity reflects the ability to limit unnecessary follow-up. Precision represents the proportion of positive predictions that were correct, and the F1-score summarizes the balance between precision and sensitivity when both false-positive and false-negative predictions are clinically relevant.

To quantify statistical uncertainty, 95% confidence intervals were estimated using 5,000 iterations of non-parametric patient-level bootstrap resampling of the pooled outer-test predictions. The 117 patient-level outer-test prediction records were sampled with replacement. For each resampled patient record, the true label, predicted probability, and previously fixed fold-specific threshold were retained. The 2.5th and 97.5th percentiles of the bootstrap distributions were reported as the confidence-interval bounds.

## 3 Results

### 3.1 Classification performance

Each patient contributed to an outer-test fold exactly once and received one prediction from the corresponding fold-specific model. Configuration, checkpoint, and threshold selection were completed without access to that patient or outer-test fold.

Across 117 pooled outer-test patient-level predictions, nested cross-validation yielded an AUROC of 0.708 (95% CI, 0.610–0.802) and an AUPRC of 0.626 (95% CI, 0.493–0.755). Using fold-specific thresholds selected exclusively from the corresponding validation data, accuracy was 0.726 (95% CI, 0.641–0.803), sensitivity was 0.694 (95% CI, 0.558–0.822), and specificity was 0.750 (95% CI, 0.643–0.853). Precision was 0.667 (95% CI, 0.532–0.796), and the F1 score was 0.680 (95% CI, 0.563–0.780).

Performance varied across the five outer folds. Mean AUROC was 0.777 ± 0.132 (range, 0.637–0.984), and mean AUPRC was 0.762 ± 0.198. Mean accuracy, sensitivity, specificity, and F1 score were 0.728 ± 0.128, 0.714 ± 0.103, 0.768 ± 0.211, and 0.689 ± 0.116, respectively. This between-fold variability reflects the uncertainty associated with the modest cohort size and supports cautious interpretation of both the fold-level and pooled performance estimates.

The pooled outer-test ROC and precision–recall curves are shown in **Figures 5** and **6**.

**Figure 5:**
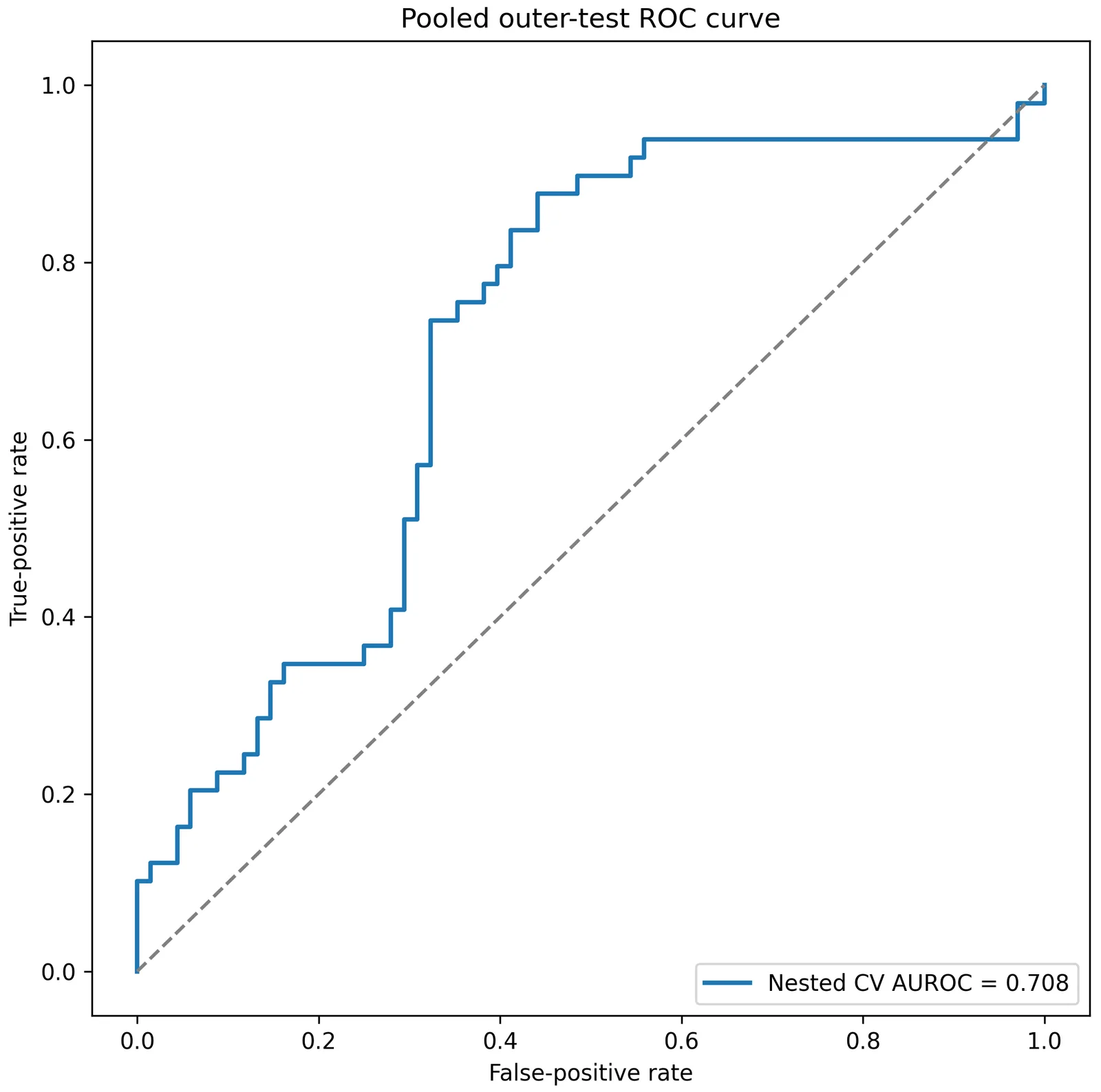
Receiver operating characteristic curve from pooled untouched patient-level outer-test predictions in five-fold nested cross-validation. The AUROC was 0.708 (95% CI, 0.610–0.802).

**Figure 6:**
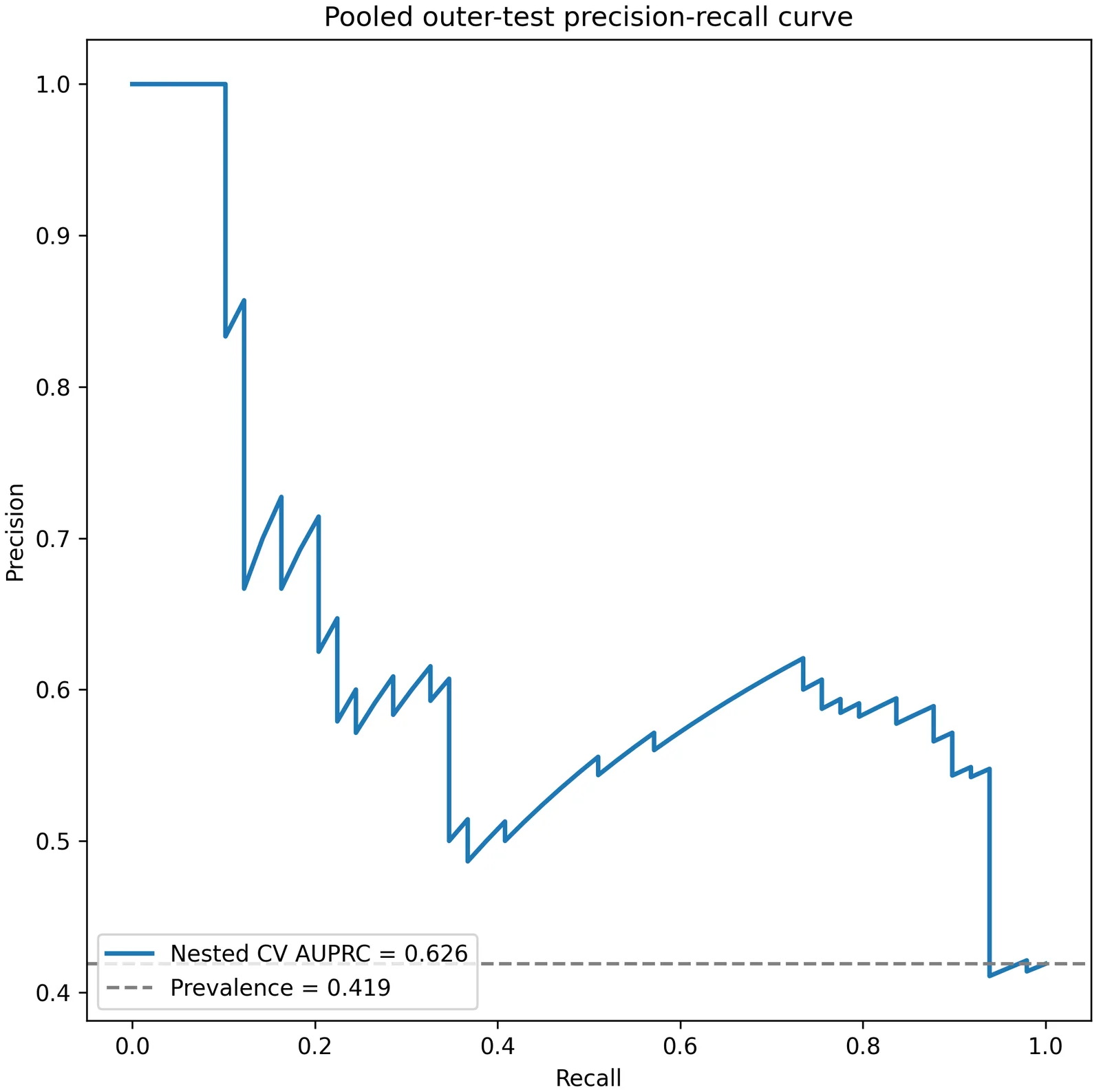
Precision–recall curve from pooled untouched patient-level outer-test predictions in five-fold nested cross-validation. The AUPRC was 0.626 (95% CI, 0.493–0.755); positive-class prevalence was approximately 0.42 (49 of 117 patients).

At the primary patient level, CME was present in 49 of 117 patients, giving a positive-class prevalence baseline of approximately 0.42 for the precision–recall analysis. The observed pooled outer-test AUPRC of 0.626 (95% CI, 0.493–0.755) exceeded this baseline, indicating moderate positive-class ranking performance.

At the validation-derived, fold-specific thresholds, the model correctly classified 85 of 117 patients: 34 true positives, 15 false negatives, 51 true negatives, and 17 false positives (**Figure 7**). The selected operating points produced a relatively balanced performance profile, with a sensitivity of 0.694 and specificity of 0.750, although clinically relevant false-positive and false-negative errors remained.

**Figure 7:**
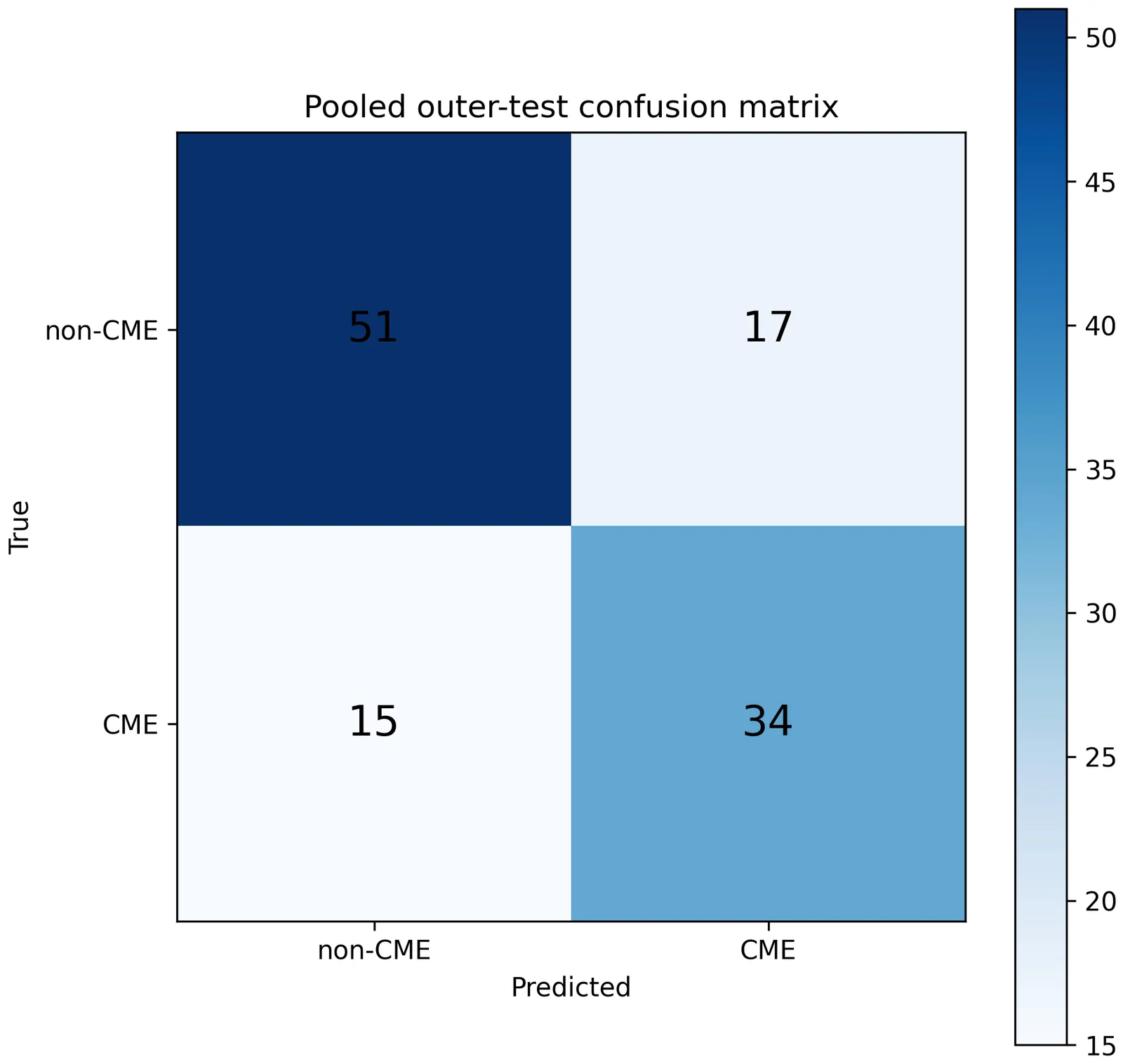
Confusion matrix from 117 pooled untouched patient-level outer-test predictions in nested cross-validation, using fold-specific thresholds selected exclusively from final-validation data.

### 3.2 Internal baseline comparison

To assess the contribution of ImageNet initialization and model architecture, we conducted a separate development-only internal benchmark after completing hyperparameter selection. Within each outerdevelopment cohort, the selected ImageNet-pretrained ResNet-18 configuration was fitted again in a separate training execution, beginning from the same ImageNet-pretrained weights and using the same four patient-level inner-validation folds. A randomly initialized ResNet-18 and a simple CNN were also trained on these folds. Both baseline models inherited the selected learning rate, scheduler, input resolution, and loss function, but were trained with all layers trainable and were not independently hyperparameter-tuned. No outer-test data were used in this comparison.

The original selection-stage evaluations of the five selected pretrained configurations produced cohortlevel mean inner-validation AUROCs of 0.902, 0.898, 0.900, 0.941, and 0.910, corresponding to 0.910 ± 0.018 across cohorts, as reported in **Table 3**. When the same selected pretrained configurations were fitted again during the separate internal benchmark, the corresponding values were 0.826, 0.893, 0.829, 0.910, and 0.888, yielding 0.869 ± 0.039. The randomly initialized ResNet-18 achieved 0.818 ± 0.024, and the simple CNN achieved 0.772 ± 0.013. Thus, the values reported in **Table 3** and those reported for the internal benchmark were obtained from separate training executions rather than from the same fitted models.

**Table 3:** Configurations selected independently within each outer-development cohort and corresponding performance across the five outer folds of nested patient-level cross-validation.

A. Configurations selected by inner cross-validation							
Fold		Learning rate	Scheduler		Input size	Fine-tuning	Loss function
1		10^−3^	Step decay		320 px	Layer3 + Layer4 + FC	Weighted cross-entropy
2		10^−3^	None		320 px	Layer3 + Layer4 + FC	Weighted cross-entropy
3		10^−3^	Cosine annealing		224 px	Layer3 + Layer4 + FC	Weighted cross-entropy
4		10^−3^	Step decay		224 px	Layer4 + FC	Weighted focal loss
5		10^−4^	None		320 px	Full network	Weighted cross-entropy
B. Inner-validation and outer-test performance							
Fold	Inner AUROC	AUROC	AUPRC	Acc.	Sens.	Spec.	F1
1	0.902	0.807	0.901	0.708	0.600	0.889	0.720
2	0.898	0.637	0.480	0.542	0.667	0.467	0.522
3	0.900	0.984	0.977	0.870	0.778	0.929	0.824
4	0.941	0.698	0.796	0.826	0.667	0.929	0.750
5	0.910	0.759	0.656	0.696	0.857	0.625	0.632
Mean *±* SD	0.910 ± 0.018	0.777 ± 0.132	0.762 ± 0.198	0.728 ± 0.128	0.714 ± 0.103	0.768 ± 0.211	0.689 ± 0.116

Panel A reports the configuration selected by maximum mean four-fold inner-validation AUROC. Panel B reports that selection AUROC and performance on the corresponding untouched outer-test fold.

The same patient partitions, fold-specific hyperparameters, and nominal random-seed schedule were used for the original and repeated pretrained-model executions. However, deterministic PyTorch algorithms were not enforced for Apple MPS; consequently, the separate executions were not guaranteed to follow identical optimization trajectories despite reuse of the same seeds. Fold-level results, configurations, and random seeds are provided in **Table 4**.

**Table 4:** Reconciliation of selection-stage and separately repeated development-only benchmark results.

A. Fold-level inner-validation AUROC.					
Outer-development		Selection-stage	Repeated pretrained	Randomly initialized	Simple CNN
cohort		pretrained ResNet-18	ResNet-18 benchmark	ResNet-18	benchmark
1		0.902	0.826	0.828	0.778
2		0.898	0.893	0.802	0.777
3		0.900	0.829	0.784	0.787
4		0.941	0.910	0.841	0.752
5		0.910	0.888	0.833	0.765
Mean *±* SD		**0**.**910** ± **0**.**018**	0.869 ± 0.039	0.818 ± 0.024	0.772 ± 0.013
B. Fold-specific configurations.					
Cohort	Learning rate	Scheduler	Input	Pretrained fine-tuning	Loss function
1	10^−3^	Step decay	320 px	Layer3 + Layer4 + FC	Weighted cross-entropy
2	10^−3^	None	320 px	Layer3 + Layer4 + FC	Weighted cross-entropy
3	10^−3^	Cosine annealing	224 px	Layer3 + Layer4 + FC	Weighted cross-entropy
4	10^−3^	Step decay	224 px	Layer4 + FC	Weighted focal loss
5	10^−4^	None	320 px	Full network	Weighted cross-entropy
C. Random seeds					
Cohort	Inner-split seed	Model-fitting seeds for inner folds 1–4			
1	1042	10142, 10242, 10342, 10442			
2	2042	20142, 20242, 20342, 20442			
3	3042	30142, 30242, 30342, 30442			
4	4042	40142, 40242, 40342, 40442			
5	5042	50142, 50242, 50342, 50442			

Panel A reports mean AUROC across four inner-validation folds per outer-development cohort. Selectionstage values correspond to the configurations reported in **Table 3** and **Supplementary Table S1**. Benchmark values came from separate development-only fits using the configurations and seed schedule shown in Panels B and C; the pretrained models began from ImageNet weights, whereas the two randomly initialized baselines were trained with all layers trainable. No outer-test data were used. Because deterministic algorithms were not enforced for Apple MPS, repeated executions were not guaranteed to be bitwise identical.

Within the separate development-only benchmark, the repeated ImageNet-pretrained ResNet-18 achieved a higher mean inner-validation AUROC than the two internal baselines. However, the pretrained configuration had been selected using these inner-validation folds, whereas the baseline models were not independently tuned. These results therefore do not establish architecture-level superiority or provide an independent estimate of generalization. The primary generalization estimate remains the pooled untouched outer-test performance reported in **Table 5**.

**Table 5:** Pooled performance of the complete model-selection pipeline on 117 untouched outer-test patient predictions from five-fold nested patient-level cross-validation.

Metric	Estimate	95% CI
Accuracy	0.726	0.641–0.803
AUROC	0.708	0.610–0.802
AUPRC	0.626	0.493–0.755
Sensitivity	0.694	0.558–0.822
Specificity	0.750	0.643–0.853
Precision	0.667	0.532–0.796
F1-score	0.680	0.563–0.780

Fine-tuning depth was examined separately within the ImageNet-pretrained candidate grid. For each candidate configuration, performance was first averaged across its four inner-validation folds. Across the resulting candidate–outer-cohort means, mean inner-validation AUROC was 0.797 ± 0.073 with fullnetwork fine-tuning, 0.805 ± 0.078 when layer3, layer4, and the classification layer were trainable, and 0.796 ± 0.085 when training was restricted to layer4 and the classification layer. Training only the final classifier resulted in substantially lower performance (0.597 ± 0.097). Thus, the three strategies that fine-tuned at least layer4 performed similarly, whereas classifier-only training produced markedly lower development-stage performance. These descriptive results were obtained from configurations evaluated on shared inner-validation partitions and should not be interpreted as independent inferential comparisons.

These analyses were restricted to model-development data and are therefore presented as exploratory evidence supporting the architecture and transfer-learning strategy rather than as independent estimates of generalization. Generalization performance is reported only from the untouched outer-test predictions of the complete nested model-selection pipeline.

### 3.3 Nested hyperparameter analysis

To characterize the influence of the principal model-development choices, we analyzed the candidate configurations evaluated within the inner loops of the nested cross-validation framework. The prespecified candidate space comprised all 144 combinations of three learning rates (10^−5^, 10^−4^, and 10^−3^), three learning-rate schedulers (none, step decay, and cosine annealing), two input resolutions (224 and 320 pixels), four fine-tuning strategies (classification layer only, layer4 and the classification layer, layer3, layer4, and the classification layer, and full-network fine-tuning), and two loss functions (weighted focal loss and weighted cross-entropy). This constituted a full factorial candidate search rather than a one-factor-at-a-time experiment.

**Table 6** summarizes marginal inner-validation performance by factor level. For each candidate within each outer-development cohort, performance was first averaged across the four inner-validation folds. These candidate–outer-cohort means were then grouped according to the factor levels present in each complete configuration. The table reports the mean and SD across the resulting candidate–outer-cohort values. Because configurations within an outer-development cohort shared inner-validation partitions and each factor level occurred with multiple combinations of the remaining factors, these results are descriptive and should not be interpreted as statistically independent or causal comparisons. Each candidate configuration was evaluated using four-fold patient-grouped inner cross-validation within each of the five outer-development cohorts. Within each outer fold, candidates were ranked according to mean inner-validation AUROC, and the highest-ranking candidate was selected for final training. Complete configuration-level results from the final 117-patient analysis for all 144 candidates in each of the five outer-development cohorts are provided in **Supplementary Table S1**. For each candidate, **Supplementary Table S1** reports the four inner-fold AUROCs, mean inner-validation AUROC, sample SD across the four inner folds, rank within the corresponding outer-development cohort, and selection status.

**Table 6:** Marginal inner-validation performance by factor level across the nested factorial candidate search.

Factor	Level	AUROC	AUPRC	Accuracy
Learning rate	10−5	0.666 ± 0.099	0.643 ± 0.098	0.724 ± 0.057
Learning rate	10−4	0.763 ± 0.128	0.742 ± 0.127	0.782 ± 0.081
Learning rate	**10**−**3**	**0**.**818** ± **0**.**078**	**0**.**793** ± **0**.**091**	**0**.**816** ± **0**.**059**
Scheduler	Cosine annealing	0.743 ± 0.122	0.719 ± 0.124	0.770 ± 0.080
Scheduler	None	**0**.**760** ± **0**.**120**	**0**.**737** ± **0**.**124**	**0**.**781** ± **0**.**076**
Scheduler	Step decay	0.744 ± 0.122	0.722 ± 0.121	0.772 ± 0.074
Input resolution	224 px	0.734 ± 0.126	0.710 ± 0.127	0.765 ± 0.079
Input resolution	**320** px	**0**.**764** ± **0**.**114**	**0**.**741** ± **0**.**117**	**0**.**784** ± **0**.**073**
Fine-tuning	Classifier only	0.597 ± 0.097	0.591 ± 0.097	0.685 ± 0.055
Fine-tuning	Layer4 + FC	0.796 ± 0.085	0.769 ± 0.102	0.804 ± 0.060
Fine-tuning	Layer3 + Layer4 + FC	**0**.**805** ± **0**.**078**	**0**.**777** ± **0**.**093**	**0**.**806** ± **0**.**058**
Fine-tuning	Full network	0.797 ± 0.073	0.767 ± 0.091	0.802 ± 0.052
Loss function	Weighted focal loss	0.743 ± 0.121	0.719 ± 0.123	0.770 ± 0.075
Loss function	Weighted cross-entropy	**0**.**755** ± **0**.**122**	**0**.**733** ± **0**.**124**	**0**.**779** ± **0**.**078**

For each candidate configuration within each outer-development cohort, performance was first averaged across its four inner-validation folds. Values are the mean ± SD of these candidate–outer-cohort means for all complete configurations containing the specified factor level. Each learning-rate and scheduler level contributed 240 candidate–outer-cohort observations, each resolution and loss-function level contributed 360 observations, and each fine-tuning level contributed 180 observations. These observations are not statistically independent because configurations within an outer-development cohort shared inner-validation partitions. The results are therefore descriptive model-development summaries and should not be interpreted as inferential comparisons, isolated causal effects, or independent estimates of generalization. Bold values indicate the highest mean within each factor.

### 3.4 Model Interpretability using Explainable AI

Gradient-weighted Class Activation Mapping (Grad-CAM) (13) was used as an exploratory method to visualize regions contributing to the patient-level CME score. For each illustrated outer-test patient, the eye producing the greatest image-level CME evidence within the patient bag was selected. The activation map was generated from the final residual block of the corresponding foldspecific ResNet-18 model.All four maps targeted the CME output class, including the true-negative and false-negative examples. Therefore, warmer colors indicate regions contributing positively to the CME score, even when the final patient-level probability remained below the fold-specific validation-derived threshold. One example from each prediction category was selected *post hoc* for qualitative illustration in **Figure 8**.

**Figure 8:**
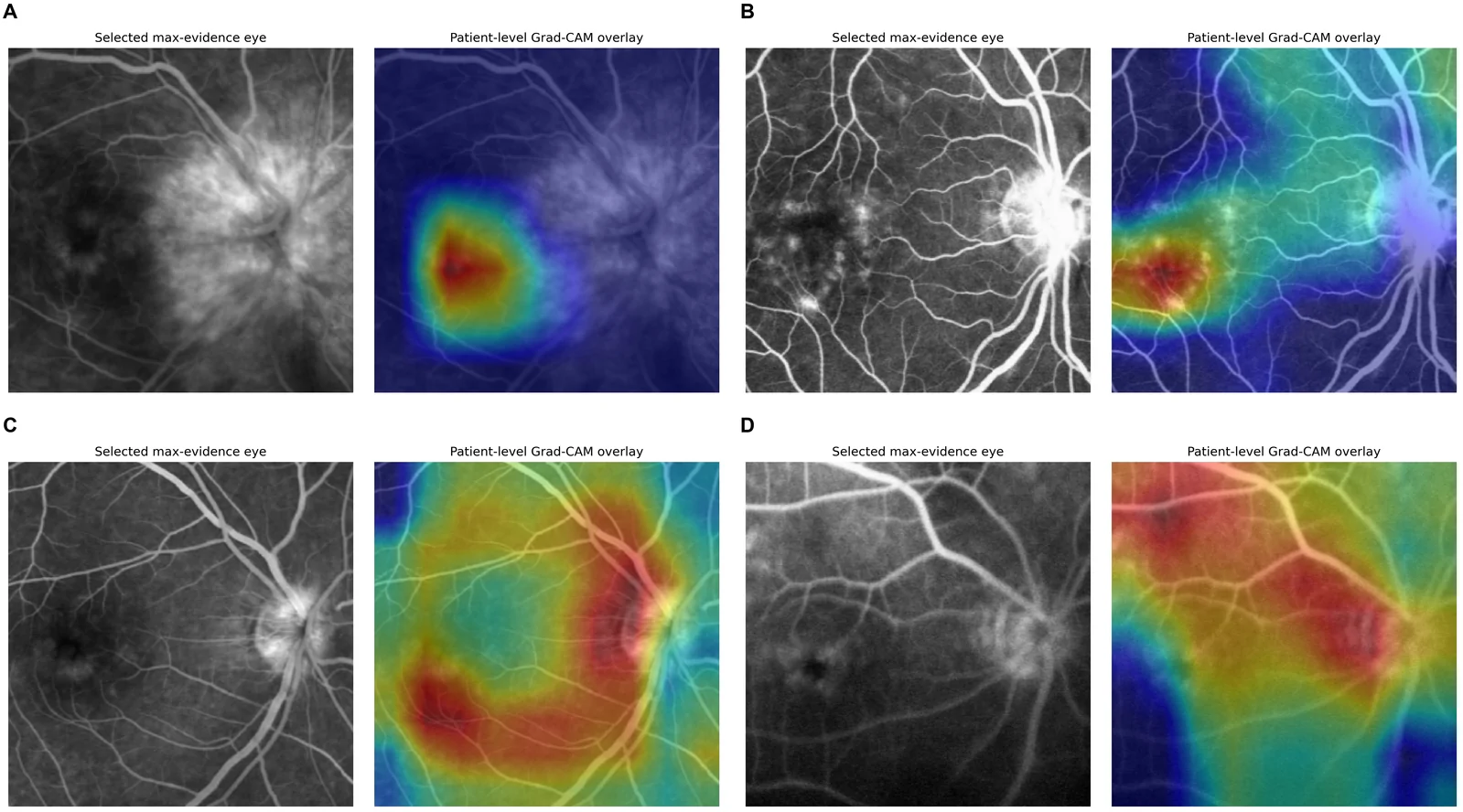
Illustrative Grad-CAM visualizations from four patient-level outer-test predictions: **(A)** true positive, **(B)** true negative, **(C)** false positive, and **(D)** false negative. For each patient, the eye producing the maximum image-level CME evidence was selected, and Grad-CAM was generated for the CME output class using the corresponding fold-specific model. Each pair shows the selected preprocessed eye and its activation overlay. Warmer colors indicate regions contributing positively to the CME score, including in the true-negative and false-negative panels. These examples were selected *post hoc* for qualitative illustration and do not constitute validated lesion localization.

In the true-positive example (**Figure 8A**), the strongest activation formed a relatively localized hotspot over the macular and perifoveal region, overlapping areas of angiographic hyperfluorescence. Activation over the optic disc and the remainder of the posterior pole was comparatively limited. This spatial distribution is anatomically plausible for a correctly classified CME-positive patient however, the overlap does not demonstrate that the model localized a specific CME lesion.

In the true-negative example (**Figure 8B**), the strongest CME-target activation was also located in the temporal macular or perimacular region, where several focal areas of hyperfluorescence were visible. More diffuse, lower-intensity activation extended across portions of the posterior pole. Despite this apparently plausible central localization, the patient-level CME probability remained below the fold-specific threshold. Because this map targets the CME class, it shows the strongest residual evidence for CME rather than the features supporting a separate non-CME classification. The example also demonstrates that macular localization alone is not specific for CME.

The false-positive example (**Figure 8C**) showed broad and multifocal activation rather than a discrete macula-centered hotspot. Prominent activation involved the peripapillary region and retinal vascular structures, with an additional region of activation extending toward the inferior-temporal image boundary. These extramacular and peripapillary patterns may have provided non-specific evidence that increased the CME score above the validation-derived threshold. This example illustrates that strong Grad-CAM activation can occur over vascular or image-region features that are not specific for CME.

In the false-negative example (**Figure 8D**), the strongest activation involved the optic-disc and peripapillary region, together with a separate broader area in the superior-temporal retina. The central macular region demonstrated comparatively less focused activation. The resulting patient-level CME probability was close to, but remained below, the fold-specific threshold. This pattern is compatible with the model assigning CME-related evidence to broader extramacular features without producing sufficiently discriminative evidence for a positive patient-level classification. Nevertheless, Grad-CAM alone cannot determine why the final probability fell below the threshold.

Collectively, these examples demonstrate substantial heterogeneity in the spatial distribution of model activation. Both the true-positive and true-negative examples contained macular or perimacular activation, whereas the false-positive and false-negative examples showed broader peripapillary, vascular, and extramacular activation. Thus, activation location did not have a simple relationship with classification correctness. These visualizations should be regarded as qualitative maps of regions associated with the CME score rather than validated localizations of CME, vascular leakage, or causal model reasoning. Formal assessment using clinician-defined annotations and a larger prespecified sample would be required to evaluate localization accuracy.

## 4 Future directions

The present study provides proof-of-concept evidence that deep transfer learning can identify angiographic patterns associated with concurrent cystoid macular edema (CME) on fluorescein angiography (FA). Because retinal vasculitis is a rare disease with inherently limited training data, the proposed transfer learning framework was specifically designed to maximize performance while reducing the risk of overfitting in this data-constrained setting. Although the nested model-selection pipeline achieved moderate discriminative performance, its current role should be regarded as a proof-of-concept rather than a clinically deployable decision support system. The next stage of research should therefore focus on improving the generalizability, reliability, and clinical applicability of the proposed methodology through larger datasets, multimodal integration, and prospective validation.

A primary priority is external validation using independent multi-center cohorts acquired across different institutions and patient populations. Although nested patient-level cross-validation provides a leakagecontrolled estimate of the internal generalization of the complete model-selection procedure, it cannot fully account for differences in image acquisition, disease prevalence, referral patterns, or patient characteristics across institutions. Validation on geographically and clinically diverse datasets will be essential to establish the reliability and reproducibility of the proposed framework before clinical translation. External validation would also permit evaluation of a single prespecified model configuration trained on the complete development cohort, which was not possible using the present dataset alone.

Another promising direction is the development of multimodal predictive models that integrate FA with complementary ophthalmic imaging modalities. Fluorescein angiography primarily captures vascular leakage and retinal perfusion abnormalities, whereas optical coherence tomography (OCT) provides highresolution structural information regarding retinal thickness and intraretinal fluid accumulation. In routine ophthalmic practice, these imaging modalities provide complementary information and are interpreted together when evaluating patients with retinal vasculitis. Future deep learning frameworks capable of jointly learning from FA, OCT, OCT angiography, color fundus photography, and relevant clinical variables may provide more comprehensive representations of disease pathology and improve both diagnostic sensitivity and specificity.

Beyond multimodal imaging, longitudinal disease modeling represents another important research direction. The present study classifies the presence of CME from a single FA examination. Future studies should investigate whether sequential imaging acquired during clinical follow-up can be used to model disease progression and predict the subsequent development or recurrence of CME. Such longitudinal models could support earlier intervention, individualized monitoring, and more personalized treatment strategies for patients with retinal vasculitis.

The findings of this study also highlight the importance of data-centric model development for rare ophthalmic diseases. The nested hyperparameter analysis indicated that learning rate and fine-tuning depth were the most influential model-development factors, whereas differences associated with scheduler, input resolution, and loss function were comparatively modest. Variation among the configurations selected across outer folds further suggests that no single complete configuration was consistently optimal in this cohort. As collaborative retinal imaging repositories continue to expand, future studies should investigate standardized annotation protocols, federated learning approaches, and larger multi-institutional datasets to improve model robustness while preserving patient privacy.

Label quality and transparency regarding diagnostic criteria are particularly critical, as interobserver variability and multimodal diagnostic dependence are common in real-world clinical settings. In the present study, ground truth labels were established retrospectively by a single uveitis specialist through review of clinical records and OCT findings. While this reflects routine clinical practice, labels were not independently assigned by multiple masked graders, and intergrader agreement could therefore not be assessed. Incorporating independent masked expert grading and formal evaluation of intergrader reliability would strengthen the reference standard and improve the robustness of model development and evaluation.

Additionally, our patient-level score is aggregated using max-pooling across available eyes, resulting in variable bag sizes between patients with unilateral versus bilateral imaging. While max-pooling reflects clinical diagnostic practice where pathology in either eye dictates patient-level management, this variation in instance count may introduce subtle disparities in patient-level probability estimation.

Model interpretability remains another critical area for future investigation. Although exploratory Grad-CAM visualizations suggested that model activations sometimes overlapped angiographic regions considered clinically relevant, these observations do not establish causal feature use or clinical validity. Future studies should evaluate whether attention maps correspond to established pathological biomarkers and investigate whether interpretable deep learning models can improve clinician confidence, facilitate error analysis, and support safe clinical implementation.

Ultimately, the greatest clinical value of artificial intelligence in retinal imaging may lie not in replacing clinician judgment but in augmenting it. Rather than serving as an autonomous diagnostic tool, the proposed framework may eventually function as part of a comprehensive clinical decision support system that assists ophthalmologists by identifying examinations with angiographic patterns associated with CME, prioritizing potentially positive examinations for specialist review. Continued collaboration among ophthalmologists, biomedical engineers, and computer scientists will be essential to translate these computational advances into clinically meaningful improvements in patient care.

## 5 Conclusion

This study provides proof-of-concept evidence that transfer learning can identify angiographic patterns associated with concurrent cystoid macular edema in patients with idiopathic retinal vasculitis. In five-fold nested, patient-level cross-validation, the complete model-selection pipeline achieved a pooled outer-test AUROC of 0.708 and AUPRC of 0.626. At validation-derived, fold-specific thresholds, accuracy was 0.726, sensitivity was 0.694, and specificity was 0.750. The operating profile was relatively balanced, with slightly higher specificity than sensitivity; 34 CME-positive patients and 51 CME-negative patients were correctly classified, while 15 false-negative and 17 false-positive errors remained.

The nested design ensured that configuration, checkpoint, and threshold selection were completed without access to the corresponding outer-test fold, providing a more defensible internal estimate of the complete development pipeline. Inner-validation analyses favored a learning rate of 10^−3^ and substantial fine-tuning of the pretrained backbone, whereas differences associated with scheduler, input resolution, and loss function were comparatively modest. Variation in the configurations selected across outer folds further indicated that no single hyperparameter configuration was consistently optimal in this cohort.

Exploratory Grad-CAM analyses suggested that model activations frequently overlapped retinal regions considered clinically relevant to CME and retinal vasculitis; however, these visualizations do not establish causal feature use or clinical validity. Overall, the findings support continued investigation of FA-based deep learning as a potential adjunctive decision-support approach for FA interpretation rather than a standalone diagnostic system. The modest single-center cohort, moderate discrimination, residual classification errors, retrospective design, and absence of external validation preclude clinical deployment. Independent multicenter validation, prospective evaluation, and integration with OCT and relevant clinical variables are required to establish generalizability and clinical utility.

## Data Availability

The datasets presented in this article are not readily available because of patient privacy, consent, and institutional restrictions. Requests to access the datasets should be directed to Celina Wang, celina.wang2027@gmail.com.

## Footnotes

1 https://github.com/celinaw09/RV_CME_ML/commit/31cedd89fb5135bb42546585b7b31614bc74df10.
